# Potential geographic distribution of the tiger mosquito *Aedes albopictus* (Skuse, 1894) (Diptera: Culicidae) in current and future conditions for Colombia

**DOI:** 10.1371/journal.pntd.0008212

**Published:** 2021-05-11

**Authors:** Emmanuel Echeverry-Cárdenas, Carolina López-Castañeda, Juan D. Carvajal-Castro, Oscar Alexander Aguirre-Obando

**Affiliations:** 1 Escuela de Investigación en Biomatemáticas, Universidad del Quindío, Armenia, Quindío, Colombia; 2 Programa de Biología, Universidad del Quindío, Armenia, Quindío, Colombia; 3 Programa de Biología Aplicada, Universidad Surcolombiana, Neiva, Huila, Colombia; 4 Instituto de Investigación de Recursos Biológicos Alexander von Humboldt, Bogotá D.C, Colombia; 5 Department of Biological Sciences, St. John’s University, Queens, New York, United States of America; Universita degli Studi di Pavia, ITALY

## Abstract

In Colombia, little is known on the distribution of the Asian mosquito *Aedes albopictus*, main vector of dengue, chikungunya, and Zika in Asia and Oceania. Therefore, this work sought to estimate its current and future potential geographic distribution under the Representative Concentration Paths (RCP) 2.6 and 8.5 emission scenarios by 2050 and 2070, using ecological niche models. For this, predictions were made in MaxEnt, employing occurrences of *A*. *albopictus* from their native area and South America and bioclimatic variables of these places. We found that, from their invasion of Colombia to the most recent years, *A*. *albopictus* is present in 47% of the country, in peri-urban (20%), rural (23%), and urban (57%) areas between 0 and 1800 m, with Antioquia and Valle del Cauca being the departments with most of the records. Our ecological niche modelling for the currently suggests that *A*. *albopictus* is distributed in 96% of the Colombian continental surface up to 3000 m (p < 0.001) putting at risk at least 48 million of people that could be infected by the arboviruses that this species transmits. Additionally, by 2050 and 2070, under RCP 2.6 scenario, its distribution could cover to nearly 90% of continental extension up to 3100 m (≈55 million of people at risk), while under RCP 8.5 scenario, it could decrease below 60% of continental extension, but expand upward to 3200 m (< 38 million of people at risk). These results suggest that, currently in Colombia, *A*. *albopictus* is found throughout the country and climate change could diminish eventually its area of distribution, but increase its altitudinal range. In Colombia, surveillance and vector control programs must focus their attention on this vector to avoid complications in the national public health setting.

## Author summary

In Colombia, little is known about the Asian mosquito distribution, *Aedes albopictus*, potential vector of arbovirus in América. Therefore, this work searched to estimate its current and future geographical distribution (under the climate change scenarios RCP 2.6 and RCP 8.5) for 2050 and 2070, using ecological niche models. We worked with models in MaxEnt, using *A. albopictus* occurrences and 8 bioclimatic variables. From its invasion to Colombia, this vector was reported in 15 departments until 1800 m. Our estimations for the currently suggest that this vector could be distributed in 96% of continental Colombia until 3000 m (≈48 million of people in infection risk). Also, for 2050 and 2070, under the RCP 2.6 scenario, its distribution could cover about near to 90% of continental Colombia until 3100 m (≈55 million of people in risk), while under RCP 8.5 scenario, could decrease below 60% of continental Colombia until 3200 m (<38 million of people in risk). In Colombia, the vigilance and vectorial control programs should focus its attention on this vector to avoid complications at the national public health level.

## Introduction

The tiger mosquito, *Aedes albopictus* (Skuse, 1894) (Diptera: Culicidae), presents vector competence for at least 26 arboviruses and some filarial nematode worms [1,2]. In continents, like Asia and Oceania, *A*. *albopictus* is the main vector for dengue, chikungunya, and Zika [3–6]. For this, in America it is not considered the prime vector for these arboviruses; however, sporadically, it has been found infected naturally with dengue in countries, like the United States (North America), Colombia, and Brazil (South America) [7–9]. Additionally, the tiger mosquito could share abiotic requirements with *Aedes aegypti* [10,11], the primary vector for dengue, chikungunya, and Zika in this continent, and whose presence in Colombia encompasses 90% of the territory up to 2300 m [12]. Currently, for these three arboviruses, no efficient vaccines exist yet [13–15]. In Colombia, historically the dengue has been the most prevalent arbovirus, for example, between 2010 and 2016, it was registered more of 674,000 cases. However, since 2014 and 2015, it was registered cases of chikungunya (690,000 cases representing more than 50% in América) and Zika (88,000 cases) in the country [16,17]. Up to now, all these arboviruses continue to circulate around the country [18].

The tiger mosquito is native to tropical, subtropical, and temperate forests of Asia and the islands of the western Pacific [19]. In these zones, favorable conditions for its development for the aquatic immature phases are estimated at water temperatures between 26 and 32 °C, while the adults require environmental temperature ranging between 25 and 31 °C and relative humidity > 70%. In addition, it has been detected in temperatures out of range 17–40 °C its survival is notably affected [20–22]. In unfavorable environmental conditions, this species presents the diapause phenomenon (diminished metabolism to very low rates of energy expenditure and subsequent inactivity) in the development of its eggs, which has permitted its dispersal at latitudes with temperate and seasonal climates, beyond its range of native distribution [23–25]. This invasion has been largely facilitated by human activities, like passive transport via maritime, land, or air cargo [26]. For the above, it has been suggested that *A*. *albopictus* exposes high ecological plasticity, considered among the 100 most invasive species in the world [26,27].

Chronologically, regarding its global invasion, *A*. *albopictus* was first registered outside its native distribution range in Europe, specifically in Albania in 1979 ([28]. Thereafter, the first populations of this species were registered in America; initially, in the center, in Trinidad and Tobago in 1983 [29], then in the north, in the United States in 1985 [30], and in the south, in Brazil in 1986 [31]. In this last part of the continent, particularly in Colombia, the tiger mosquito was first registered in Leticia (Amazon, on the border with Tabatinga, Brazil) in 1998, in a suburban area with abundant vegetation [32]. Since then, it has been registered in 52 locations of 12 departments of the 32 that make up the country [33]. In Colombia, however, there are some areas where *A*. *albopictus* presence is still unknown and given its vector competence, therefore, it is necessary to recognize it in order to include it in the Vector Borne Diseases and Zoonoses program and the futures Public Health Plans [34,35].

One way of complementing the lack of knowledge of the distribution of *A*. *albopictus* in Colombia is through ecological niche modeling (ENM). This tool enables characterizing the fundamental niche of a species and then estimating its potential geographic distribution from presence records and environmental variables [36–39]. Given the relevance of the ENM for public health, these have been used to estimate the potential distribution of mosquitoes of medical importance belonging to the *Haemagogus* [40], *Culex* [41], *Anopheles* [42] and *Aedes* genera [10,43]. Particularly for *A*. *albopictus*, its potential distribution has been estimated in Australia [44], western Europe [45], the United States [46], Mexico [47], Guatemala [48], and globally [10,49,50].

Furthermore, climate change could influence directly on the geographic distribution of invasive mosquitoes. The Intergovernmental Panel on Climate Change (IPCC) has formulated different climate scenarios, known as Representative Concentration Paths (RCP), which estimate distinct greenhouse gas emission levels and CO_2_ over time (i.e. 2050 and 2070). Among them, there is RCP 2.6 based on a gas emissions peak (~ 421 ppm), being the scenario with lowest effects on climate, and RCP 8.5 based on continuous increase of gas emissions (~ 936 ppm), considered the scenario with the most drastic climate effects [51]. Taking into consideration the different gas emission scenarios, investigations conducted until now suggest that the geographic distribution of *A*. *albopictus* could vary significantly in the long term, which would imply that the viral diseases transmitted by this vector could disperse to new places in the country and previously unaffected human populations could be exposed to contagion [10,19,26,52,53]. Due to the aforementioned, it is necessary to better understand the current distribution of *A*. *albopictus* and its likely future variations in Colombia. Therefore, this work sought to estimate and quantify the current potential geographic distribution of this vector in Colombia and identify the effect of climate change on its distribution under RCP 2.6 and 8.5 emission scenarios by 2050 and 2070 by using the ENM approach. It is hypothesized that currently, the tiger mosquito could find suitable areas for its distribution in all the departments of Colombia, while, in the future, under the effects of climate change, it could increase the suitable areas for its distribution in the departments with colder climates and decrease in warmer climates.

## Materials and methods

### Study area

The Republic of Colombia is located in northeastern South America and borders geographically with the republics of Venezuela, Brazil, Peru, Ecuador, and Panama. Additionally, it has coastal zones on the Caribbean and on the Pacific Ocean. Its continental extension is of 1.141.748 Km^2^ and its political-administrative division comprises 32 departments [54].

### Ecological niche modeling and estimation of accessible area

In eastern Asia, the native distribution for *A*. *albopictus* is concentrated in urban, semi-urban and rural areas in the biomes: tropical and subtropical rain forest, tropical and subtropical dry forest, temperate forest, and mixed forest. Starting from the aforementioned, point presence records of the tiger mosquito in its native area were used to characterize its accessible area (M) according to biotic regions (biomes) [55] [see the second sentence of data of *A*. *albopictus* presence for more details]. Then, we used two modeling methods, the spatial, where the current environmental conditions of the native distribution of tiger mosquito was projected in South America, and the spatial-temporal, where we projected and suggested a possible distribution of this vector under the effects of climate change [46,56]. From each projection in South America, estimations corresponding to the continental area of Colombia were extracted to describe the current and future potential distribution of the tiger mosquito.

### Data of *A*. *albopictus* presence

From a published literature review, reports available in the Colombian National Health Institute (CNHI) [57] and the Global Biodiversity Information Facility (GBIF) database [58] indicate that two sets of occurrence data were formed. The first, compiled the occurrences of the native range of the tiger mosquito available in the GBIF and those collected by Kamal *et al*., [10] and these were used for estimation of M and training models. The second data set correspond to invasion occurrences of *A*. *albopictus* in South America and this was used for validation of the current conditions model [56,59,60]. Of these, for occurrences in Colombia, the altitude and coverage type layers were superimposed on the map of this country, which allowed the extraction of data for altitude (m), location area (urban, semi-urban and rural) and coverage type (urban tissue (buildings made by humans), urban green area (patches of forest within a city) and open forest (natural areas)) where *A*. *albopictus* has been recorded. The types of covers and location areas, were defined following the proposal for CORINE land cover methodology established by the *Instituto de Hidrología*, *Meteorología y Estudios Ambientales* of Colombia [61]. Thus, the first and second datasets were conformed initially by 2,085 and 3,414 records, respectively. The data was screened, excluding records without spatial geo-referencing, with geo-spatial problems (a record up the ocean or in a not corresponding area to that described), duplicate presences and multiple presences in a single pixel, at a resolution of 2.5 min (~5 Km^2^) [10,46,49]. For this, the *raster* 3.0–7 [62], *rgdal* 1.4–8 [63], *dismo* 1.1–4 [64] and *usdm* 1.1–18 [65] libraries of R [66] were used. After the data filtering, the first and second datasets were consolidated with 1,328 and 3,406 occurrences, respectively (data in S1 File).

### Climate data

From the WorldClim database v. 2.0, 21 environmental variables were downloaded with a spatial resolution of 2.5 min [10,46,49], whose values are based on averaged data since 1970 to 2000 [67]. These variables were submitted to two analyses to define their inclusion in the calibration of the models. First, the contribution of each variable was determined through the Jackknife test generated in MaxEnt, maintaining those whose accumulated contribution added to 95%. Then, with the variables selected, a Spearman correlation in R was conducted. Between the variables highly correlated positively (R > 0.8) or negatively (R <– 0.8), we were discarded for the ENM those with a lower influence on the biological development of *A*. *albopictus*.

To assess the potential distribution of the species within a context of climate change, the variables resulting from prior analyses were downloaded from the Climate Change, Agriculture and Food Security—CCAFS [68] platform, with values estimated by the HadGEM2-ES model for 2050 and 2070, for RCP 2.6 and 8.5 emission scenarios. We did not consider the scenarios RCP 4.5 and RCP 6.0 once these correspond to stabilization phases between RCP 2.6 and RCP 8.5 [69], therefore, the possible projections to be obtained would correspond to intermediate phases of the scenarios studied here. The HadGEM2-ES model, developed by the Hadley Center (UK), is one of the most adequate to analyze future projections in tropical areas of South America [69–73]. All the layers of the variables selected were adjusted to the extension of M defined and from South America using QGIS v.3.4.0 [74], for its later use in the estimations described ahead.

### Geographic distribution estimations

Three contexts were proposed to analyze the potential geographic distribution of *A*. *albopictus* in Colombia, and in each its latitudinal and altitudinal variation were identified. For the first context, to training model, the first dataset was used, together with the layers of the environmental variables under the current conditions cut to the native extension. In the two remaining contexts, the potential effects of climate change were estimated on the distribution of the tiger mosquito in Colombia by 2050 and 2070, through the emission paths RCP 2.6 and 8.5 for each period, respectively. For projection of the three contexts, we used the corresponding layers cut to South American extension.

All the estimations were made through the maximum entropy algorithm, implemented in the MaxEnt software v.3.4.1 k [75]. This algorithm was used due to its high accuracy when estimating distribution areas, allowing to calibrate the models through datasets of different sizes, determining the contribution of each environmental variable in the estimations performed; it may be used for predictions in multiple spatial and temporal scales and only requires presence data to conduct the estimations [56,76,77]. For each scenario proposed, 10 replicates were executed per 1000 iterations, using a logistic output format, this included a range of 0 to 1 of presence probability. For future estimations, the parameters “*Do Clamping*” and “*Extrapolation*” were deactivated to avoid extrapolations in the extreme values of the ecological variables (non-analog climates) [10].

The estimates obtained in MaxEnt were simplified in a binary format to distinguish two areas categories: potential distribution areas and non-potential distribution areas of the tiger mosquito (continuous maps in Figs A-E of S2 File). For this, we followed the conservative least realized by Gómez-Palacio [78], where the threshold consisting in the lowest environmental suitability value corresponding to any site of occurrence, considering an omission value of 0.2 [78–81]. Finally, the potential distribution area, in pixels, was quantified in all the scenarios using QGIS’ tools and later on, we converted the values to kilometers. We subtracted the potential areas of each climate change scenario of the current estimation to know the variation.

We calculate the quantity of people in high exposition to bites of *A*. *albopictus* for all scenarios according to data of last national census, in 2018, by Departamento Administrativo Nacional de Estadística (DANE) [82], and its futures estimates of population growth in Colombia.

### Validation of the model

This work only evaluated the performance of the model under current conditions, given that the behavior of *A*. *albopictus* is unknown upon eventual future climate scenarios. To do so, the metric of the area under the curve (AUC) was considered as estimated in MaxEnt. Additionally, to obtain greater support on the performance of the model, the AUC significance level was determined through a partial analysis of the Receiver Operating Characteristics (partial ROC), employing the second dataset, previously described in data of *A*. *albopictus* presence [81,83].

For statistical significance, we performed a partial ROC test on the Niche Toolbox platform [81,84], where the E parameter was adjusted to 0.2 per 1000 iterations. As criterion to evaluate the model’s significance, it was considered that AUC values with p > 0.05 indicate that the estimations made are not better than those generated by a random model, while AUC with p < 0.05 indicates that the predictions estimated are better than those obtained from a random model [43,81].

## Results

**Fig 1:**
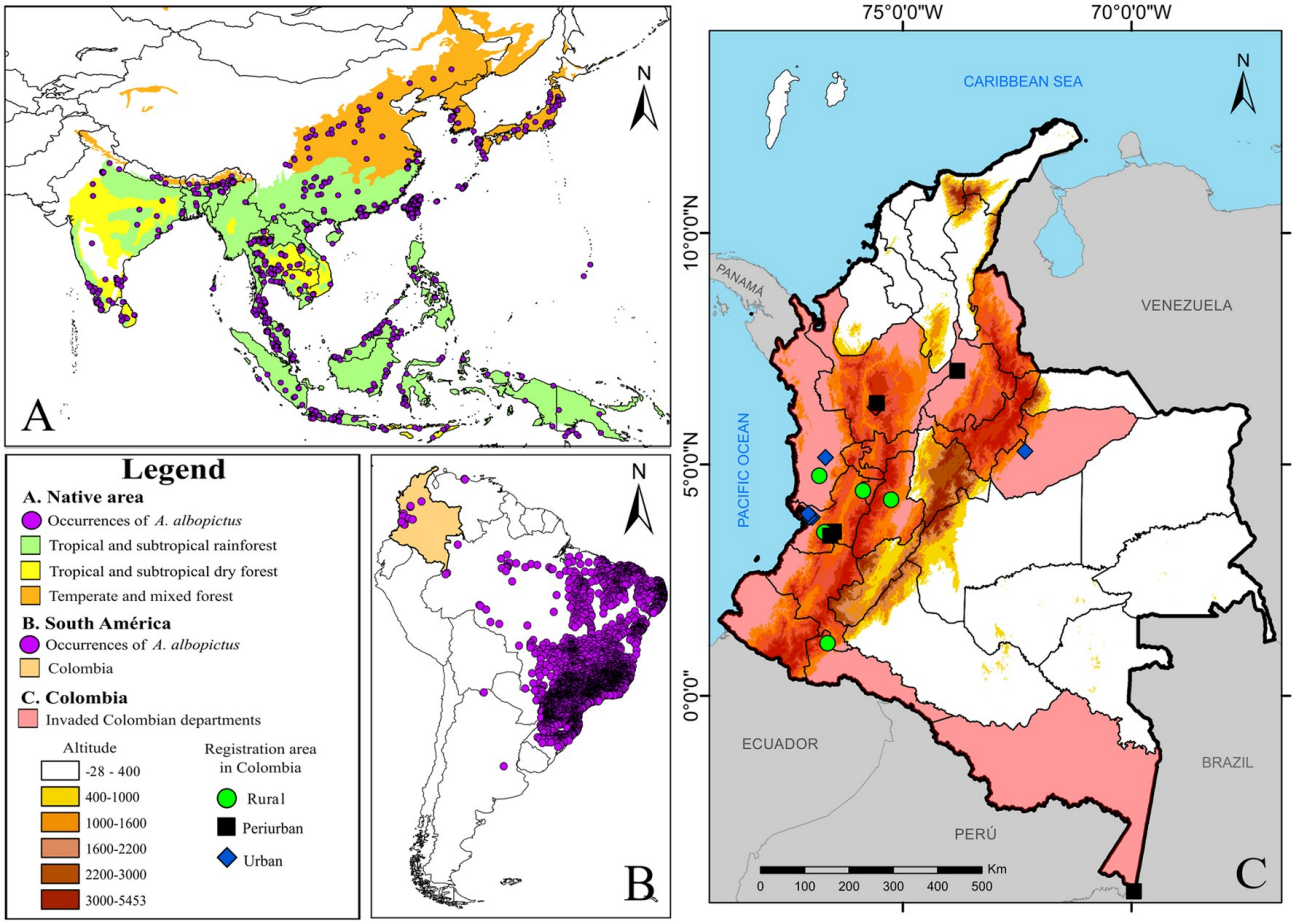
Occurrences of *A*. *albopictus* in: **A**. Native area (first dataset), **B**. South America (second dataset), and **C**. Colombia, employed in the ENM. The maps were built using the free and open source QGIS software version 3.10.11 (https://www.qgis.org/en/site/about/index.html) and shapefiles were obtained from the free and open source DIVA-GIS site (https://www.diva-gis.org/gdata).

Since the first record, in 1998 in Colombia, *A*. *albopictus* has been registered in 52 locations of 15 departments, between 0 and 1800 m. The information was gathered for 45 locations of which 27 had data about the location of the capture sites. The seven locations not collected correspond to poorly detailed CNHI information or to personal communications with other authors. The departments with more occurrences registered were Antioquia (24.5%) and Valle del Cauca (22.5%). In addition to this, the suitability habitats to the tiger mosquito were higher in urban areas (57%), followed by rural areas (23%) and peri-urban areas (20%) (Fig 1). In urban areas, the tiger mosquito has been associated principally with relicts of forests immersed in the urban matrix (Table 1). Additionally, a ENM was made using the occurrences of *A*. *albopictus* throughout the world (Figs A-E of S3 File).

**Table 1:** Records of *Aedes albopictus* in Colombia and its border limits.

Department	City/Municipality	Sampling site	Altitude (m)	Coverage type	Area of location	Year of record	Reference
Amazonas	Leticia	Carrera 9 and Calle 4 •	0	Urban green area	Semi-urban	2003	[58]
		Secretary of Health	78	Urban green area	Urban	1998	[32]
Antioquia	Bello	Piamonte natural reserve •	1515	Open forest	Semi-urban	2017	[58]
	Medellín	Olaya Herrera airport	1496	Urban tissue	Urban	2011	[85]
		CASD	1613	Urban green area	Urban	2011	
		Los Molinos shopping center •	1539	Urban tissue	Urban	2011	
		San Diego shopping center •	1486	Urban tissue	Urban	2011	
		Used tire distributor	1465	Urban tissue	Urban	2011	
		Botanical Garden •	1467	Urban green area	Urban	2011	
		Gilberto Alzate Avendaño School	1509	Urban green area	Urban	2011	
		Market place	1514	Urban green area	Urban	2011	
		North transport terminal	1465	Urban green area	Urban	2011	
		South transport terminal	1496	Urban tissue	Urban	2011	
	Yondó	NA	100	NA	NA	NA	[57]
Boyacá	NA	NA	NA	NA	NA	NA	[86]
Caldas	Viterbo	NA	1000	NA	NA	NA	[57]
Casanare	Yopal	Industrial zone	313	Urban green area	Urban	2016	[87]
Cauca	Bolívar	NA	1700	NA	NA	NA	[57]
Chocó	Bagadó	NA	200	NA	NA	NA	[57]
	El Cantón de San Pablo	NA	35	NA	NA	NA	[57]
	Istmina	San Agustín •	25	Open forest	Rural	2015	[88]
		Substation •	54	Urban green area	Urban	2015	
Nariño	La Unión	NA	1727	NA	NA	NA	[57]
	Samaniego	NA	1450	NA	NA	NA	[57]
	San Pablo	NA	1750	NA	NA	NA	[57]
	Sandoná	NA	1800	NA	NA	NA	[57]
	Taminango	NA	1375	NA	NA	NA	[57]
Norte de Santander	NA	NA	NA	NA	NA	NA	[86]
Putumayo	Mocoa	15 de Mayo settlement	650	NA	Rural	2017	[33]
		Nueva Esperanza settlement	650	NA	Rural	2017	
		El Porvenir neighborhood	650	NA	Rural	2017	
Quindío	La Tebaida	Vereda La Palmita •	1183	Open forest	Rural	2015	[89]
Risaralda	La Virginia	NA	900	NA	NA	NA	[57]
	Marsella	NA	1600	NA	NA	NA	[57]
	Pueblo Rico	NA	1563	NA	NA	NA	[57]
Santander	Barrancabermeja	Yariguíes airport •	120	Open forest	Semi-urban	2010	[90]
Tolima	Rovira	Vereda Boquerón •	917	Open forest	Rural	2016	[58]
Valle del Cauca	Buenaventura	Kennedy neighborhood •	6	Urban tissue	Urban	2001	[91]
		La Unión neighborhood •	19	Urban green area	Urban	2001	
		Maritime Terminal •	9	Urban tissue	Urban	2004	[58]
	Cali	Almaviva •	954	Urban green area	Urban	2006	[92]
		Aloccidente •	970	Open forest	Semi-urban	2006	
		Alpopular •	970	Open forest	Semi-urban	2006	
		La Balastrera •	1468	Open forest	Rural	2006	
		Forestry checkpoint •	1359	Open forest	Semi-urban	2006	
		Transport terminal •	977	Urban tissue	Urban	2006	
	La Cumbre	NA	1584	NA	NA	NA	[57]
	Ulloa	NA	1410	NA	NA	NA	[57]

^•^ Occurrences used in the ENM.

NA: not available

Table 2 presents the environmental variables used to calibrate the ENM, including mean annual temperature and annual precipitation variables; although a high correlation was present due to their importance in the life cycle of *A*. *albopictus*.

**Table 2:** Climate variables used in the ENM for the tiger mosquito in Colombia.

Variables	Unit
Mean annual temperature	°C
Range of daily temperatures	°C
Isothermality	%
Annual precipitation	mm
Precipitation of the rainiest month	mm
Precipitation of the driest month	mm
Precipitation of the rainiest quarter	mm
Precipitation of the warmest quarter	mm

**Fig 2:**
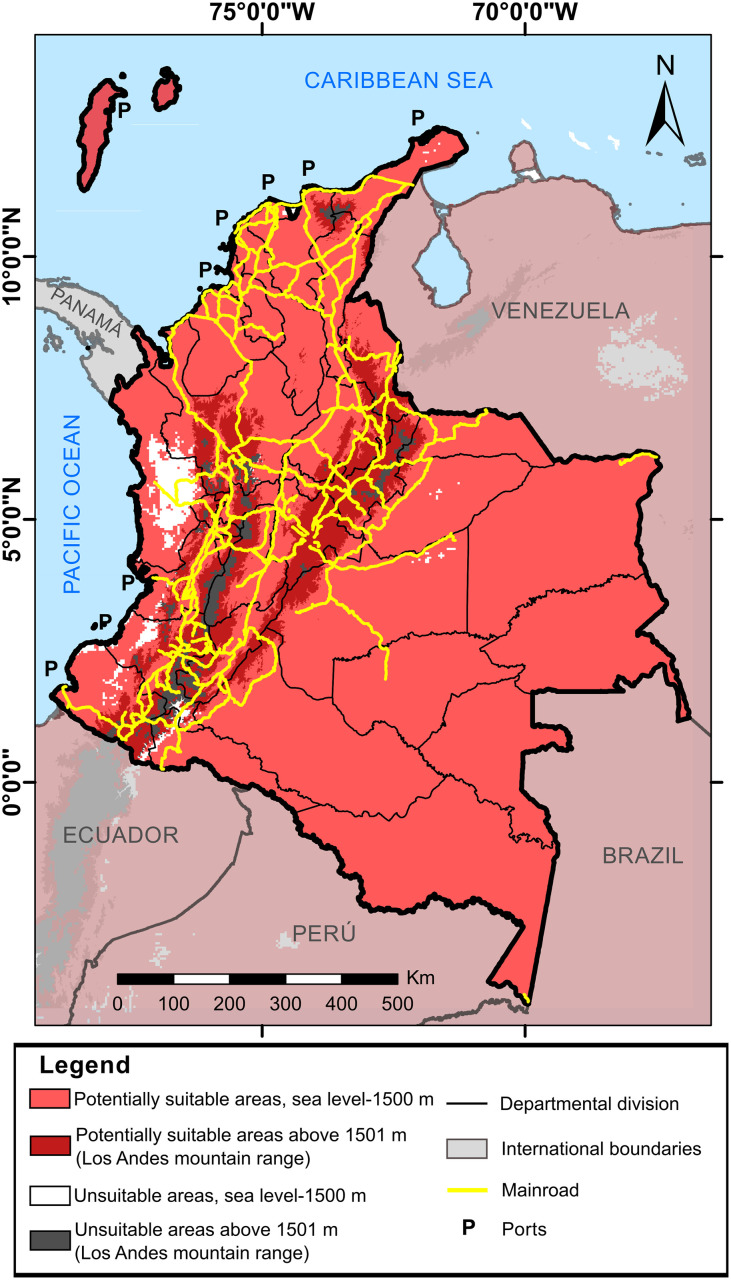
Potentially suitable areas to distribution of *A*. *albopictus* under current conditions in Colombia. The light red corresponds to binary threshold. The map was built using the free and open source QGIS software version 3.10.11 (https://www.qgis.org/en/site/about/index.html) and shapefiles were obtained from the free and open source DIVA-GIS site (https://www.diva-gis.org/gdata).

For Colombia, results of predictions of *A*. *albopictus* currently estimated its habitat suitability in 96.14% of the continental area in all the departments, including altitudes up to 3.000 m (Fig 2). The AUC metric estimated in MaxEnt was 0.9, while the partial ROC supported statistically the predictions (p < 0.001). Under this scenario, we estimated more than 48 million of people would be at risk of acquiring some of the arboviruses that the tiger mosquito could transmit (Table 3). The thresholds values for each context proposed are showing there too.

**Table 3:** Areas of current and future potential distribution of *A*. *albopictus* in Colombia.

Context	Scenario	Cut threshold	Area of potential geographic distribution (Km^2^)	Proportion of occupied area (%)	People at risk of exposure
Current		0.11859	1 098 615.9	96.14	48’426,992
2050	RCP 2.6	0.28254	1 016 000.8	88.91	55’120,670
	RCP 8.5	0.30788	684 314.2	59.88	37’125,809
2070	RCP 2.6	0.27220	1 023 652.7	89.58	56’457,738
	RCP 8.5	0.31355	296 342.5	25.93	16’344,243

**Fig 3:**
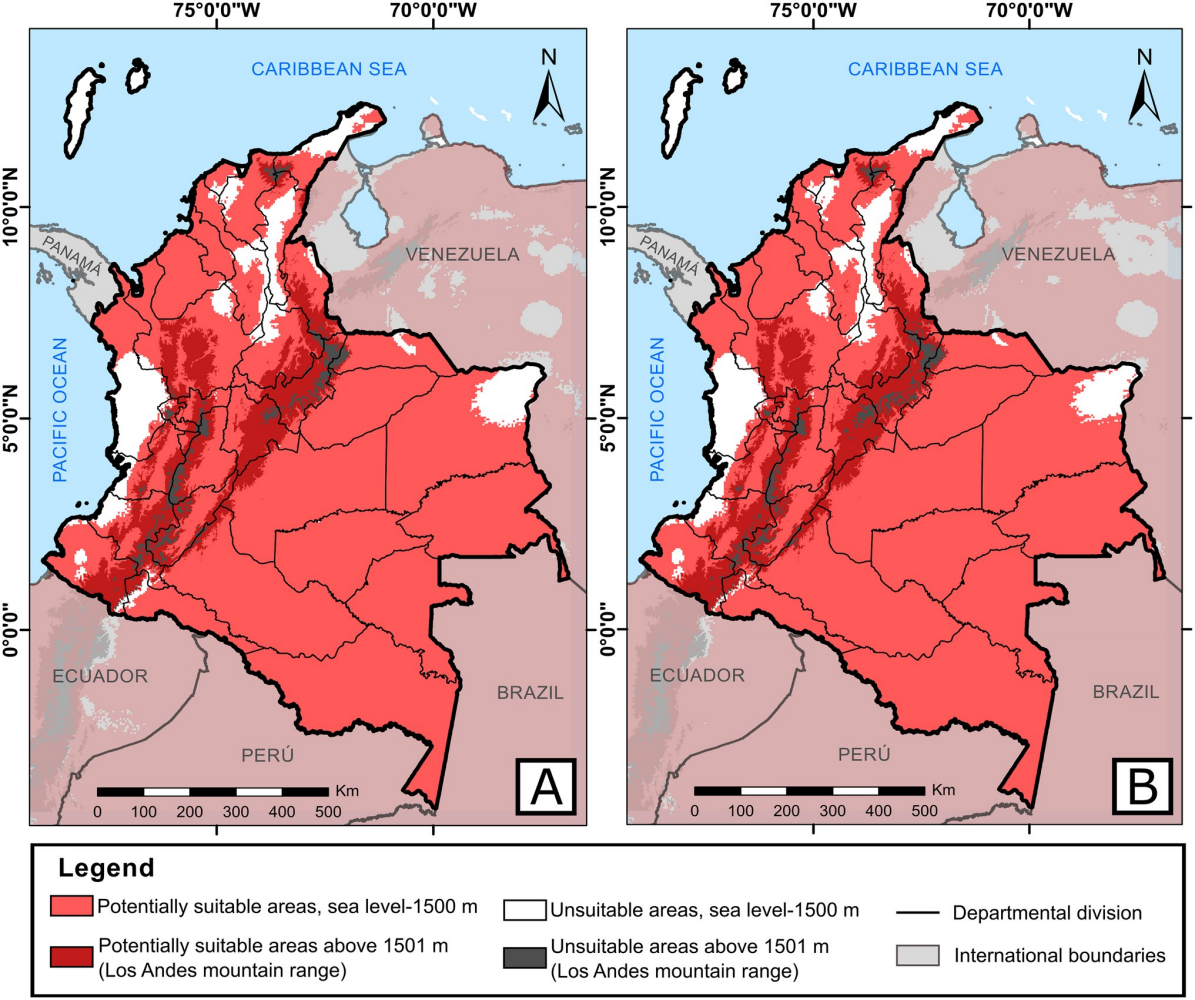
Potentially suitable areas to distribution of *A*. *albopictus* within a context of climate change for: A. 2050 and B. 2070, under the RCP 2.6 emission scenario. The light red corresponds to binary threshold. The maps were built using the free and open source QGIS software version 3.10.11 (https://www.qgis.org/en/site/about/index.html) and shapefiles were obtained from the free and open source DIVA-GIS site (https://www.diva-gis.org/gdata).

**Fig 4:**
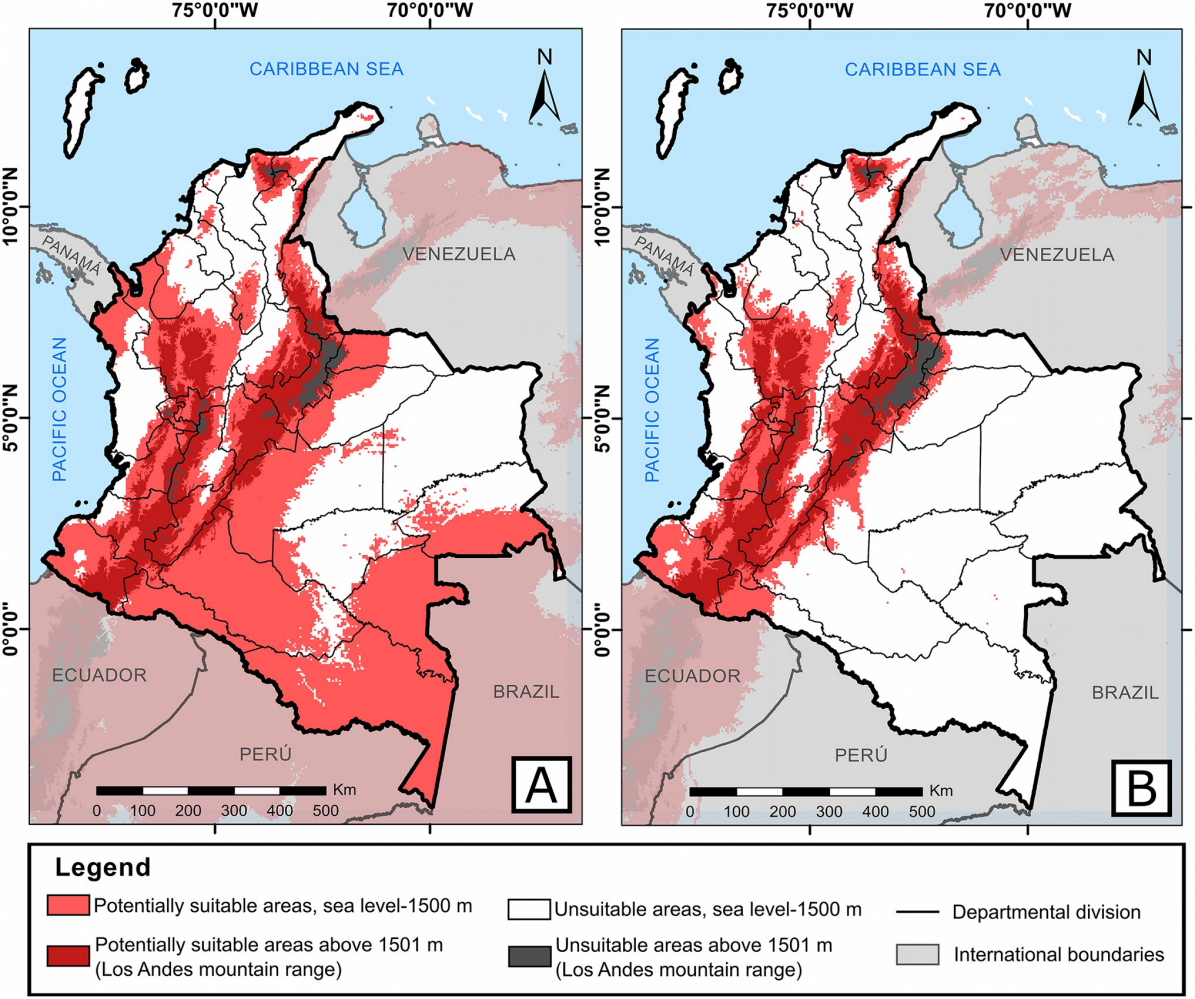
Potentially suitable areas to distribution of *A*. *albopictus* within a context of climate change for: A. 2050 and B. 2070, under the RCP 8.5 emission scenario. The light red corresponds to binary threshold. The maps were built using the free and open source QGIS software version 3.10.11 (https://www.qgis.org/en/site/about/index.html) and shapefiles were obtained from the free and open source DIVA-GIS site (https://www.diva-gis.org/gdata).

Predictions of *A*. *albopictus* within a context of climate change for 2050 and 2070 estimated that the departments of Nariño, Cauca, Huila, Quindío, Risaralda, Caldas, Cundinamarca, and Boyacá could have the same distribution observed currently. Under the RPC 2.6 emission scenario, the tiger mosquito had the same distribution pattern in which it could continue present in over 88% of the Colombian continental surface and increase its distribution range up to 3100 m for both years. For the departments of Chocó, Valle del Cauca, Cauca, Vichada, Santander, Cesar, Bolívar, La Guajira, and San Andrés y Providencia greater decrease could occur in the potential area with respect to current values (Fig 3A and 3B). The people exposed to this vector would increase between 6–8 million for both years, based on estimated population growth, taking as reference the population that could be at risk (Table 3). Additionally, under the environmental conditions of the RCP 8.5 emission scenario by 2050 and 2070, *A*. *albopictus* could eventually broaden its altitudinal range up to 3200 m. By 2050, environmental conditions could cause a decrease of its distribution and people exposed to bites (Table 3) in the departments of La Guajira, Magdalena, Atlántico, Bolívar, Sucre, Córdoba, Cesar, western Santander, eastern Norte de Santander, eastern Tolima, Chocó, western Valle del Cauca, western Cauca, Arauca, Casanare, Vichada, Meta, Guainía, and Guaviare (Fig 4A). Besides these departments, by 2070, the area of potential distribution could also diminish in peripheral zones of Antioquia, Vaupés, Caquetá, Putumayo, and Amazonas where its distribution would be restricted to the departments associated with the Andes mountain rage principally, like Nariño, central-eastern Cauca, central-eastern Valle del Cauca, Huila, western Tolima, Quindío, Risaralda, Caldas, central-southern Antioquia, Cundinamarca, Boyacá, eastern Santander, and central-western Norte de Santander, besides buffer zones of the Sierra Nevada of Santa Marta to the north of the country (Fig 4B). Therefore, people exposed could diminish too, to a few most of 16 million in all country (Table 3).

## Discussion

Current estimates suggest that *A*. *albopictus* could have broad distribution in Colombia. It has been observed that the invasion of this mosquito to other countries started in the coastal zones [93] and, thereafter, disseminated to their interior [94,95]. In this sense, we can hypothesize that ports to the Pacific Ocean of Buenaventura (Valle del Cauca), Guapi (Cauca) and Tumaco (Nariño) [96], through which 50% of commercial imports enter the country in ships, most of them from Asia (native place to *A*. *albopictus*), could have played an important role in its initial invasion to the country. Furthermore, it should not be discarded that maritime ports located on the Atlantic Ocean (Caribbean) in the departments of Sucre, Bolívar, Atlántico, Magdalena, La Guajira, and San Andrés y Providencia [97], where official reports of this vector are still not available, also could have facilitated its invasion. Added to this, climate conditions of all the coastal departments mentioned [98] are similar to the conditions registered in its native area, thereby, favoring its survival and reproduction [99]. An increase has been observed in coastal zones of cases of diseases transmitted by vectors, principally of *Anopheles* and *Aedes* genera, due to El Niño and La Niña climate phenomena, which have favored increments of artificial oviposition sites (water tanks, containers, etc.) or natural sites (plants, puddles, etc.) and, consequently, increasing the population size of the vectors and the probability of arbovirus transmission [100–104].

Upon establishing the populations of the tiger mosquito in the departments with coastal zones, land passive transport may have also played an important role in its distribution to the rest of Colombia, as noted in other parts of the world [105]. High roadway connectivity, as well as national vehicular flow between the center, west and north of the country, and international connections with western Venezuela–where records already exist of *A*. *albopictus* [106], would permit rapid invasion of the tiger mosquito to new departments [93–95,107,108].

In Santander, Antioquia, Quindío, Caldas, Risaralda, and Tolima, where the tiger mosquito has been registered in 19 locations [57,58,85,89,90], a current broad distribution was also estimated. The vast geographic and environmental heterogeneity (mix of natural and urban areas) and urban-rural transitions of these departments, similar to those of its native area, increases availability and diversity of microhabitats, as well as the number of breeding sites in which the tiger mosquito could develop its immature stages and increase quickly its population size [85]. In addition to this, the country’s human population and the 492 mammal species reported [109,110] represent potential food sources and, thereby, subsistence for the tiger mosquito [10,14]. Furthermore, in these places, *A*. *aegypti* is widely disseminated up to 2300 m, together with the circulation of dengue, chikungunya, and Zika [16,111] for which this species is the principal vector in America. Due to this, the role of *A*. *albopictus* in the transmission of these arboviruses cannot go unnoticed given the panorama mentioned and this vector should be included in surveillance and control strategies of said diseases, given that new alternatives to control *A*. *aegypti* are being implemented in this continent. Among said strategies, we can highlight the use of transgenic mosquitoes (known as Release of Insect Carrying a Dominant Lethal Gene -RIDL-; mosquitoes released seeking to eliminate the vector in a particular location) and infected with *Wolbachia pipientis*–WMel lineage (mosquitoes with refraction to arboviruses transmitted by *A*. *aegypti*) [112,113]. For this reason, if in any zone of the country with presence of both species, it was suppressed or establish populations of *A*. *aegypti* refractory for dengue, chikungunya, and Zika, the known and unknown populations of tiger mosquito for control programs, these could assume the role of the main vector of these arboviruses due to its vectorial competence since populations have been found to be naturally infected with these diseases [16,34,35,114–117]. In America the role of the tiger mosquito in the transmission of dengue, chikungunya, and Zika has been widely discussed, however, the absence of studies focused on the detection of this vector limits the identification of a better approach to the behavior in the transmission of arbovirus [9,118]. However, its vectorial capacity documented in other countries in the Americas indicates the potential vectorial role that it could play for public health [15,119]. On the other hand, in Africa and Asia, where both species inhabit [10,120], *A*. *albopictus* plays an important role in the transmission of those diseases, while in America, *A*. *aegypti* is the main vector [4–6,121]. Therefore, the potential role that the tiger mosquito can play in the transmission of those diseases in which *A*. *aegypti* is a recognized vector cannot be underestimated.

In 17 departments of north, east, southeast and low-center of Colombia, the presence of *A*. *albopictus* has not been reported; however, predictions indicate that it would also be present in such. Pabón *et al*., [122] suggested that in the departments of north, east and southeast of Colombia, where 15 of the departments without occurrence records of *A*. *albopictus* are located, the mean annual temperature can variate between 24–30 °C (±1.3 °C) and 24–28 °C (±3.3 °C), while, the annual precipitation between 500–3500 mm and 1500–5000 mm, respectively. Therefore, these lands have similar climatic conditions to those of native areas of *A*. *albopictus*, that would allow their survival and distribution [13,20–22]. Additionally, although in the departments associate to the Andean mountain range the climatic conditions can variate significatively for altitude [122], *A*. *albopictus* has established in most of them [54,57,85,89], probably, since its invasion of Colombia, it has acquired characteristics that have allowed it to expand its geographical distribution along the country [123].

In addition, increased temperature, sea level, and precipitation variability are some effects brought by climate change, therefore, some places in which now *A*. *albopictus* could be present, in the future would not have adequate conditions for its permanence [124]. Nonetheless, in mountainous zones of Colombia where temperatures are currently cold and act as an ecological restriction for invading arthropods [125], variations in temperature could favor the establishment of the tiger mosquito even in altitudes above those that have been currently registered (up to 1800 m) by 2050 (up to 3100 m) and 2070 (up to 3200 m) [23,24,57].

By 2050 and 2070, under the RCP 2.6, its distribution could decrease in some departments characterized historically by high temperatures, like Vichada and Guajira [25] and those mostly affected by El Niño phenomenon, like coastal zones (Chocó, Valle del Cauca, and Cauca), however could include altitudes of 3100 m. Under the RCP 8.5 scenario, we suggest that the environmental conditions could change drastically by 2050 and 2070 (close to 60%), which would provoke a considerable decrease in the distribution of the tiger mosquito in the country with respect to current values, as hypothesized globally [50]. In this order of ideas, this vector’s distribution could be limited in most of the departments associated with the Andes mountain range, increasing its distribution up to 3200 m, which would maintain favorable conditions for its survival.

This research provides relevant information on the distribution of *A*. *albopictus* in Colombia and each of its departments, however, we can highlight some limitations: a. These models do not take into account the effect of biotic variables, referring to interspecific relationships of the tiger mosquito [10,126]; b. the low number of georeferenced occurrences of the tiger mosquito in Colombia and the high aggregation of occurrences in South America could have caused a lower performance in the model validation statistic under current conditions; c. There are no variables on human dynamics and changes in land use, a determining factor for the establishment of vector mosquito populations, in the calibration of the models [127]; d. the absence of occurrences to evaluate the models projected into the future will always be a limitation within the ENM [10]; e. finally, despite the fact that various factors limit it, it would be ideal to carry out field trips to the estimated potential distribution sites to corroborate the results obtained.

For the future, we suggest further research with genetic evidence to identify the invasion points of *A*. *albopictus* in west and north of Colombia and the dispersion along all country and we recommend to Colombian vector control programs to make inspections in the estimate’s areas in this study in order to verify the presence of *A*. *albopictus* and to take control vector measures.

## Conclusion

From 1998 to recent years, the tiger mosquito has been detected in rural, urban and semi-urban areas. Currently, *A*. *albopictus* could be distributed in 96% of Colombia, including altitudes up to 3,000 m, being the country’s environmental conditions, the food sources, and passive transport possible key factors for its invasion to new departments where it has yet to be registered. Moreover, the effects of climate change by 2050 and 2070 could generate increase in its altitudinal range up to 3200 m and affect the presence of the tiger mosquito in the country’s coastal, plains, and jungle zones, but could remain principally in the Andean departments. For 4 of the 5 scenarios evaluated here, the human population at risk of exposure and disease transmission exceeds 35 million people. Finally, greater attention should be paid to this potential vector in Colombia, given that it has abiotic requirements as that of *A*. *aegypti*, as well as vector competence for dengue, chikungunya and Zika, diseases with elevate number of cases in the recent years, which would complicate public health in the country.

## Supporting information

S1 File *A*. *albopictus* records for the training models and validation of the scenarios. **A**. Native records; **B**. South American records; **C**. Global records.
(XLSX)

S2 File Continuous maps for the potential geographic distribution of *A*. *albopictus* in Colombia. A. current conditions; B. RCP 2.6 by 2050; C. RCP 8.5 by 2050; D. RCP 2.6 by 2070; E. RCP 8.5 by 2070. Warm areas: suitable; Cold areas: unsuitable, for tiger mosquito. The maps were built using the free and open source QGIS software version 3.10.11 (https://www.qgis.org/en/site/about/index.html) and shapefiles were obtained from the free and open source DIVA-GIS site (https://www.diva-gis.org/gdata).
(ZIP)

S3 File Geographic distribution for Colombia using world records of *A*. *albopictus*. A. current conditions; B. RCP 2.6 scenario by 2050; C. RCP 8.5 scenario by 2050; D. RCP 2.6 scenario by 2070; E. RCP 8.5 scenario by 2070. The maps were built using the free and open source QGIS software version 3.10.11 (https://www.qgis.org/en/site/about/index.html) and shapefiles were obtained from the free and open source DIVA-GIS site (https://www.diva-gis.org/gdata).
(ZIP)
