# Supplementary figures and images for: Potential geographic distribution of the tiger mosquito Aedes albopictus (Skuse, 1894) (Diptera: Culicidae) in current and future conditions for Colombia
Source: PLoS Negl Trop Dis. 2021 May 11;15(5):e0008212. doi: 10.1371/journal.pntd.0008212 (PMC8112644; doi:10.1371/journal.pntd.0008212)

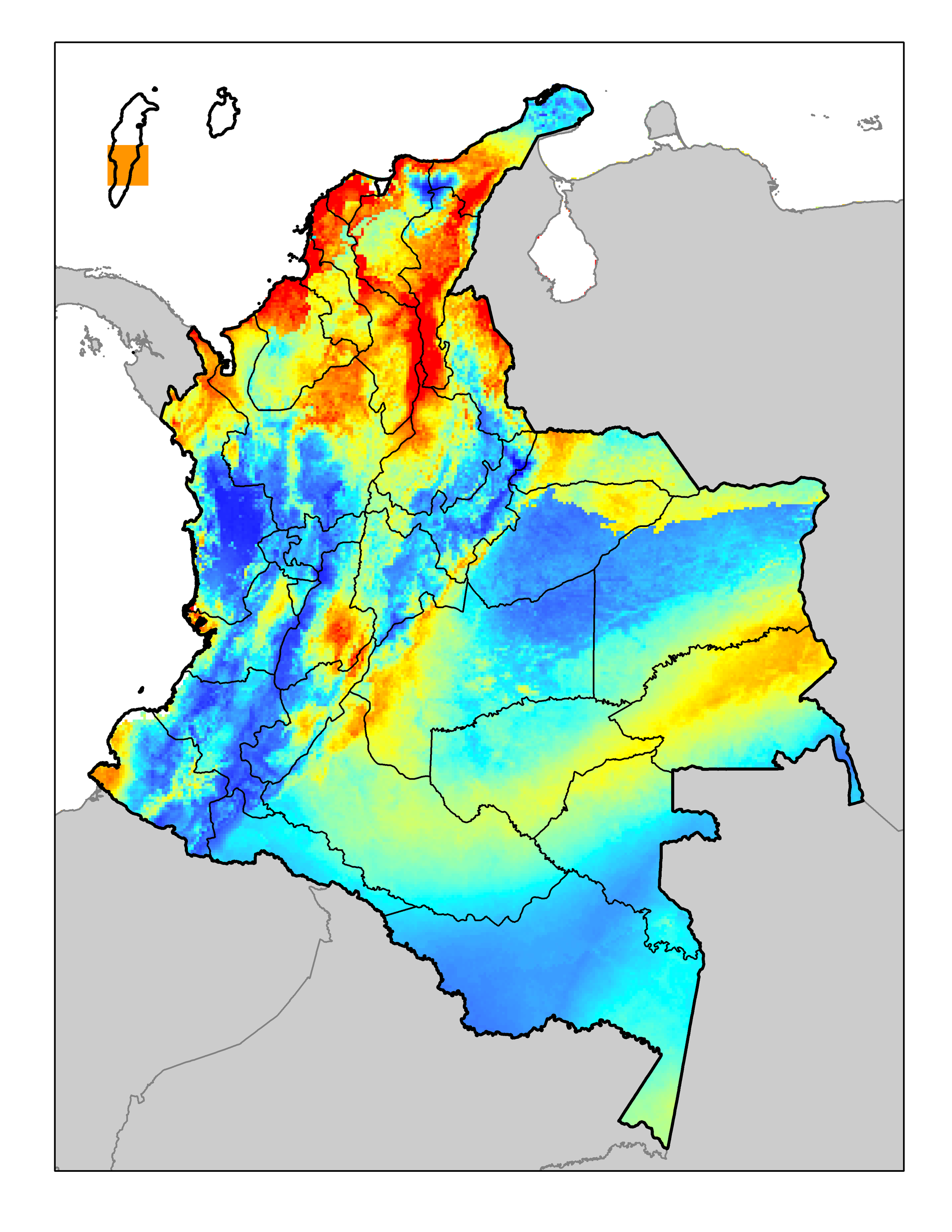

Supplement: S2 File — A. current conditions; B. RCP 2.6 by 2050; C. RCP 8.5 by 2050; D. RCP 2.6 by 2070; E. RCP 8.5 by 2070. Warm areas: suitable; Cold areas: unsuitable, for tiger mosquito. The maps were built using the free and open source QGIS software version 3.10.11 (https://www.qgis.org/en/site/about/index.html) and shapefiles were obtained from the free and open source DIVA-GIS site (https://www.diva-gis.org/gdata). (ZIP) [file pntd.0008212.s002.zip › S2_Fig_A.tif]

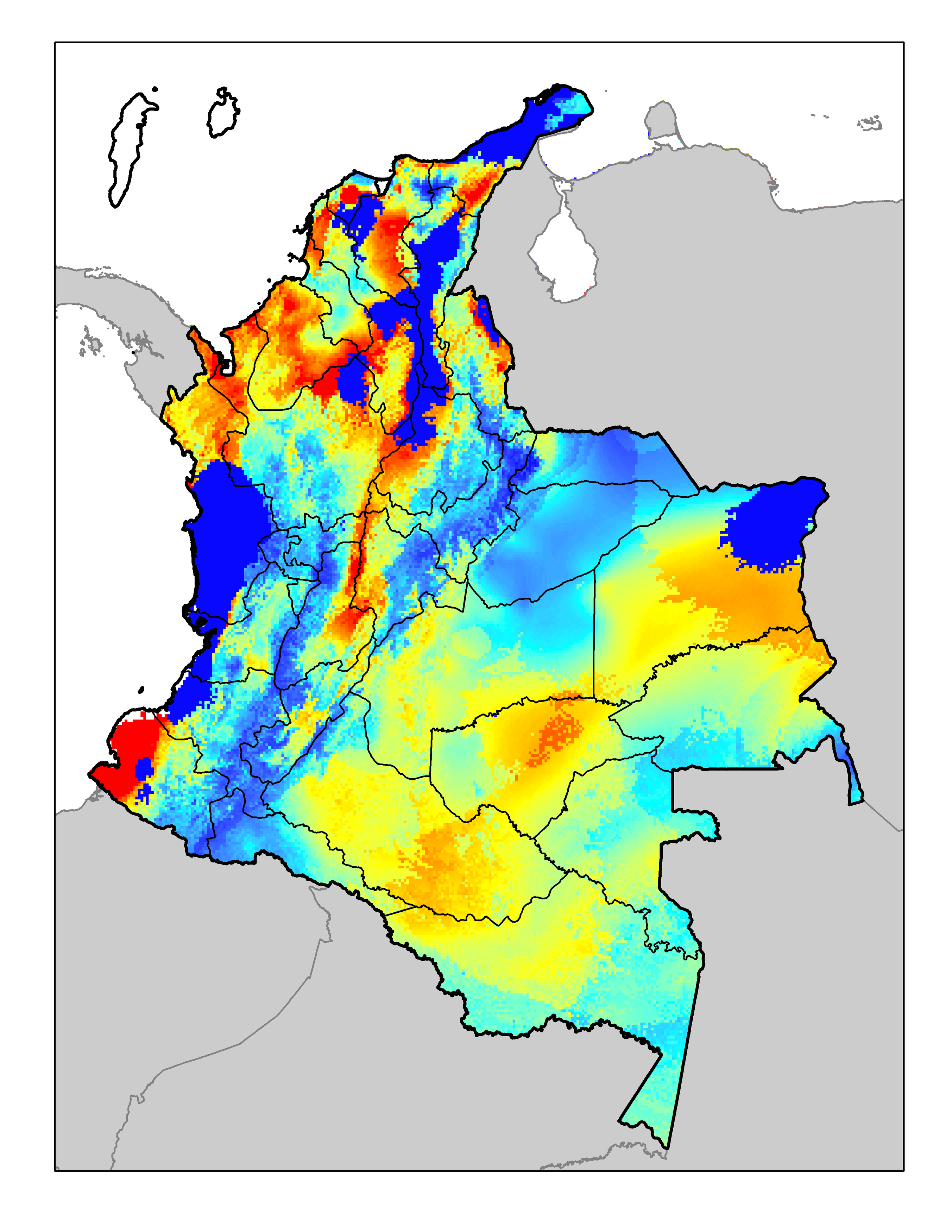

Supplement: S2 File — A. current conditions; B. RCP 2.6 by 2050; C. RCP 8.5 by 2050; D. RCP 2.6 by 2070; E. RCP 8.5 by 2070. Warm areas: suitable; Cold areas: unsuitable, for tiger mosquito. The maps were built using the free and open source QGIS software version 3.10.11 (https://www.qgis.org/en/site/about/index.html) and shapefiles were obtained from the free and open source DIVA-GIS site (https://www.diva-gis.org/gdata). (ZIP) [file pntd.0008212.s002.zip › S2_Fig_B.tif]

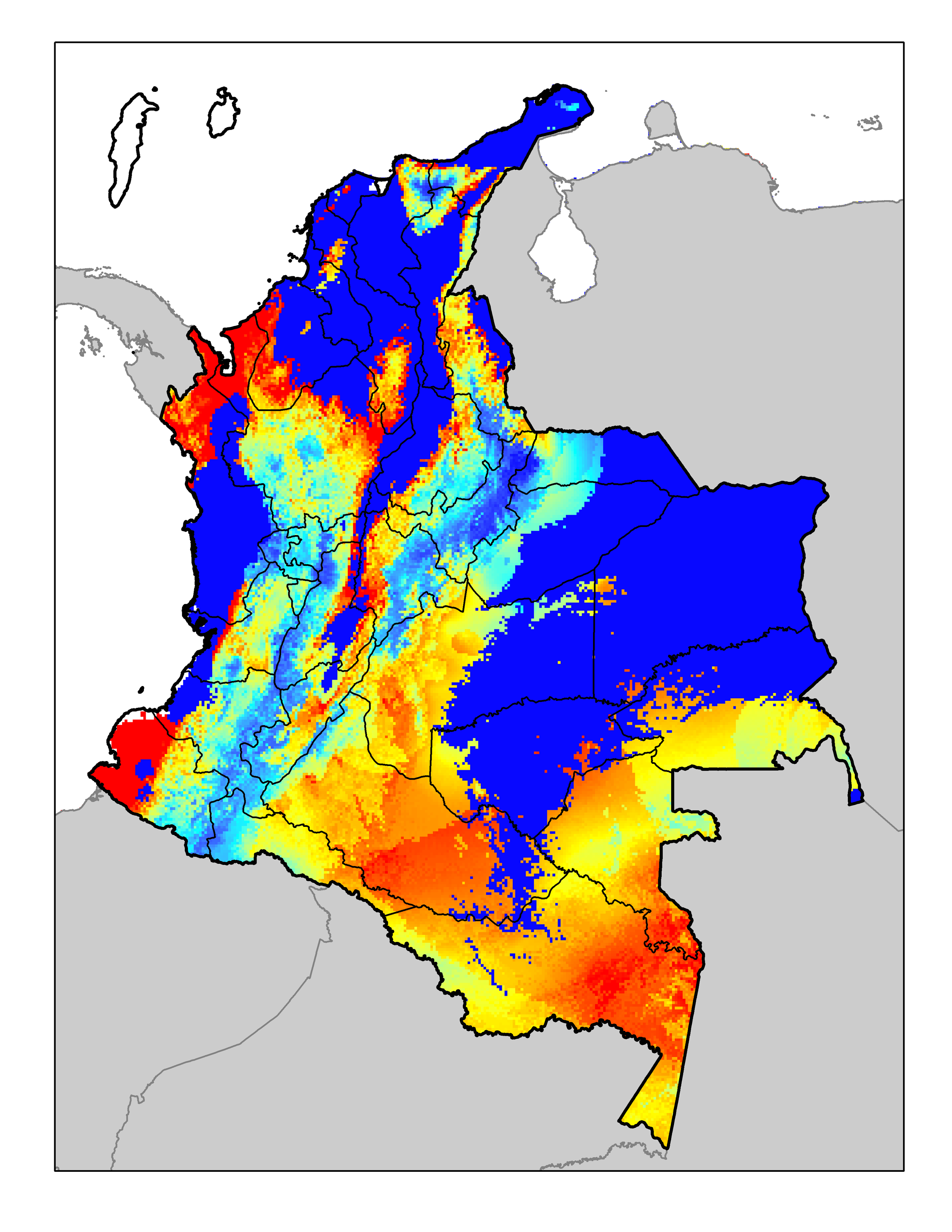

Supplement: S2 File — A. current conditions; B. RCP 2.6 by 2050; C. RCP 8.5 by 2050; D. RCP 2.6 by 2070; E. RCP 8.5 by 2070. Warm areas: suitable; Cold areas: unsuitable, for tiger mosquito. The maps were built using the free and open source QGIS software version 3.10.11 (https://www.qgis.org/en/site/about/index.html) and shapefiles were obtained from the free and open source DIVA-GIS site (https://www.diva-gis.org/gdata). (ZIP) [file pntd.0008212.s002.zip › S2_Fig_C.tif]

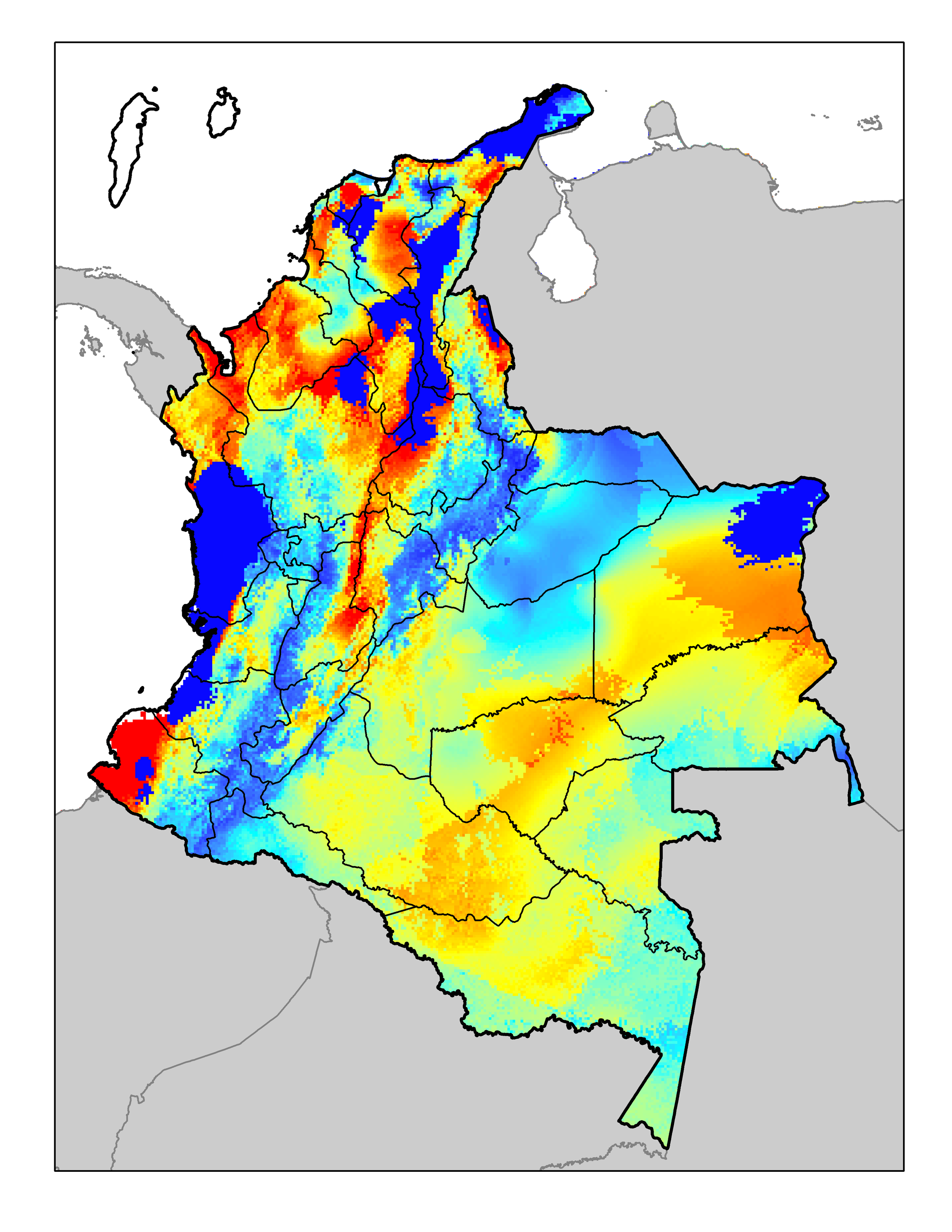

Supplement: S2 File — A. current conditions; B. RCP 2.6 by 2050; C. RCP 8.5 by 2050; D. RCP 2.6 by 2070; E. RCP 8.5 by 2070. Warm areas: suitable; Cold areas: unsuitable, for tiger mosquito. The maps were built using the free and open source QGIS software version 3.10.11 (https://www.qgis.org/en/site/about/index.html) and shapefiles were obtained from the free and open source DIVA-GIS site (https://www.diva-gis.org/gdata). (ZIP) [file pntd.0008212.s002.zip › S2_Fig_D.tif]

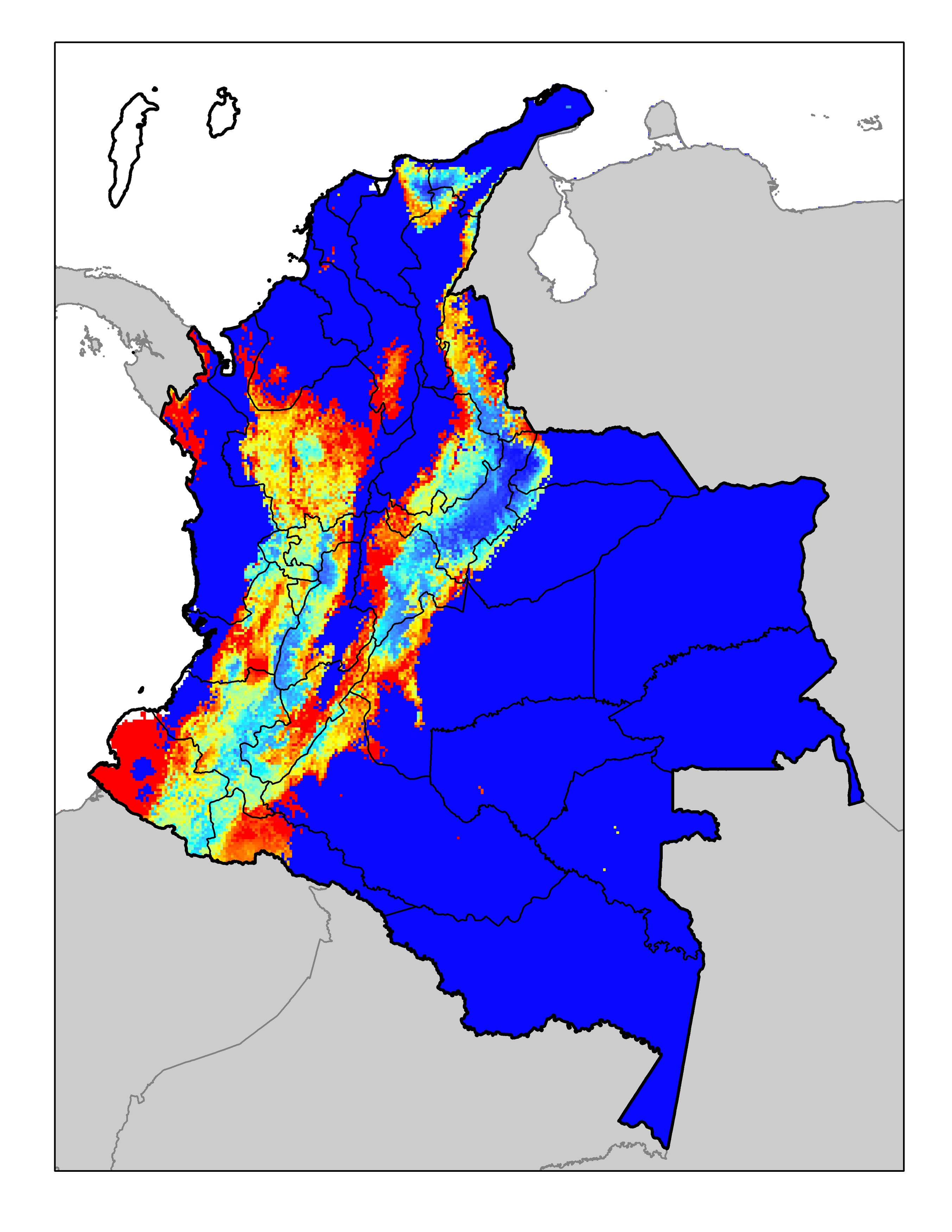

Supplement: S2 File — A. current conditions; B. RCP 2.6 by 2050; C. RCP 8.5 by 2050; D. RCP 2.6 by 2070; E. RCP 8.5 by 2070. Warm areas: suitable; Cold areas: unsuitable, for tiger mosquito. The maps were built using the free and open source QGIS software version 3.10.11 (https://www.qgis.org/en/site/about/index.html) and shapefiles were obtained from the free and open source DIVA-GIS site (https://www.diva-gis.org/gdata). (ZIP) [file pntd.0008212.s002.zip › S2_Fig_E.tif]

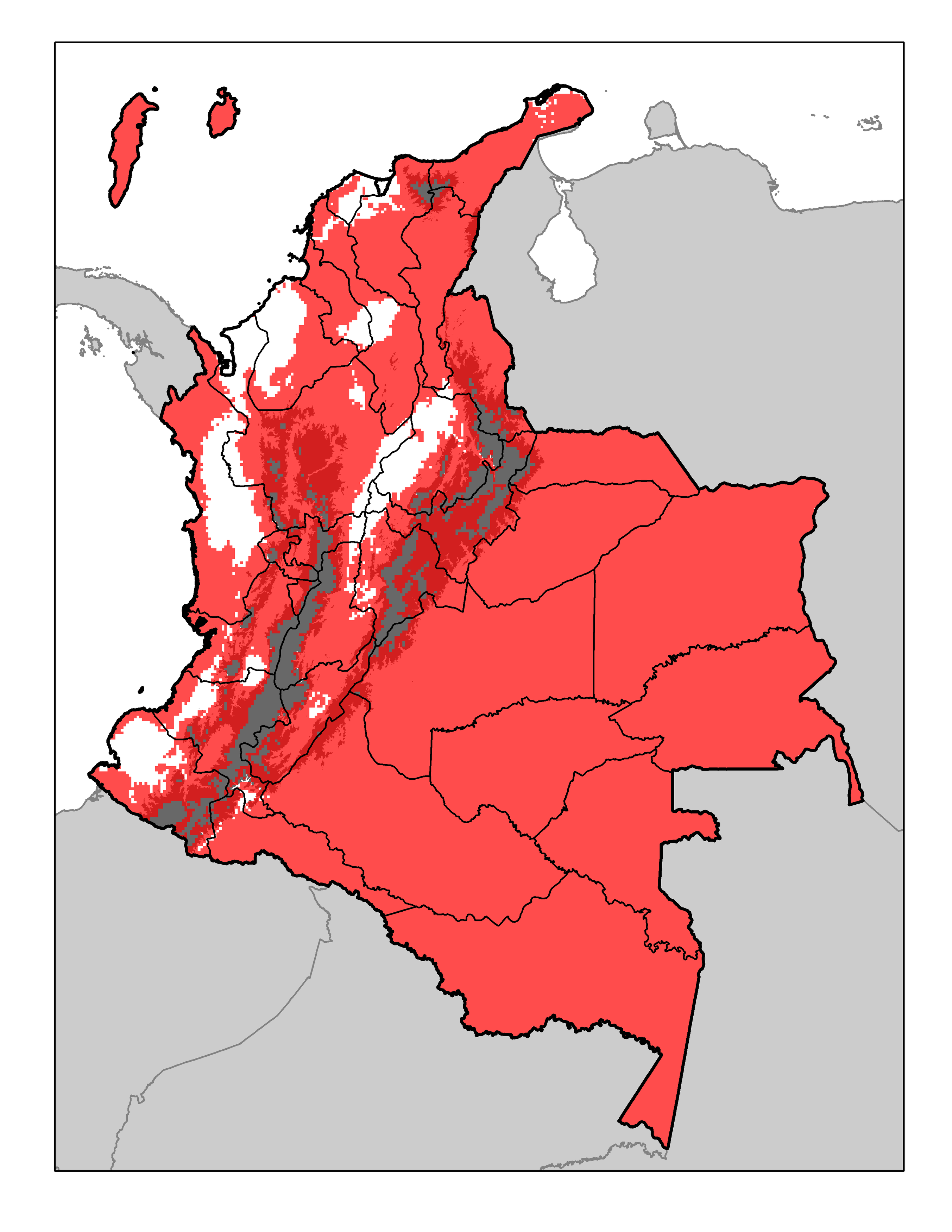

Supplement: S3 File — A. current conditions; B. RCP 2.6 scenario by 2050; C. RCP 8.5 scenario by 2050; D. RCP 2.6 scenario by 2070; E. RCP 8.5 scenario by 2070. The maps were built using the free and open source QGIS software version 3.10.11 (https://www.qgis.org/en/site/about/index.html) and shapefiles were obtained from the free and open source DIVA-GIS site (https://www.diva-gis.org/gdata). (ZIP) [file pntd.0008212.s003.zip › S3_Fig_A.tif]

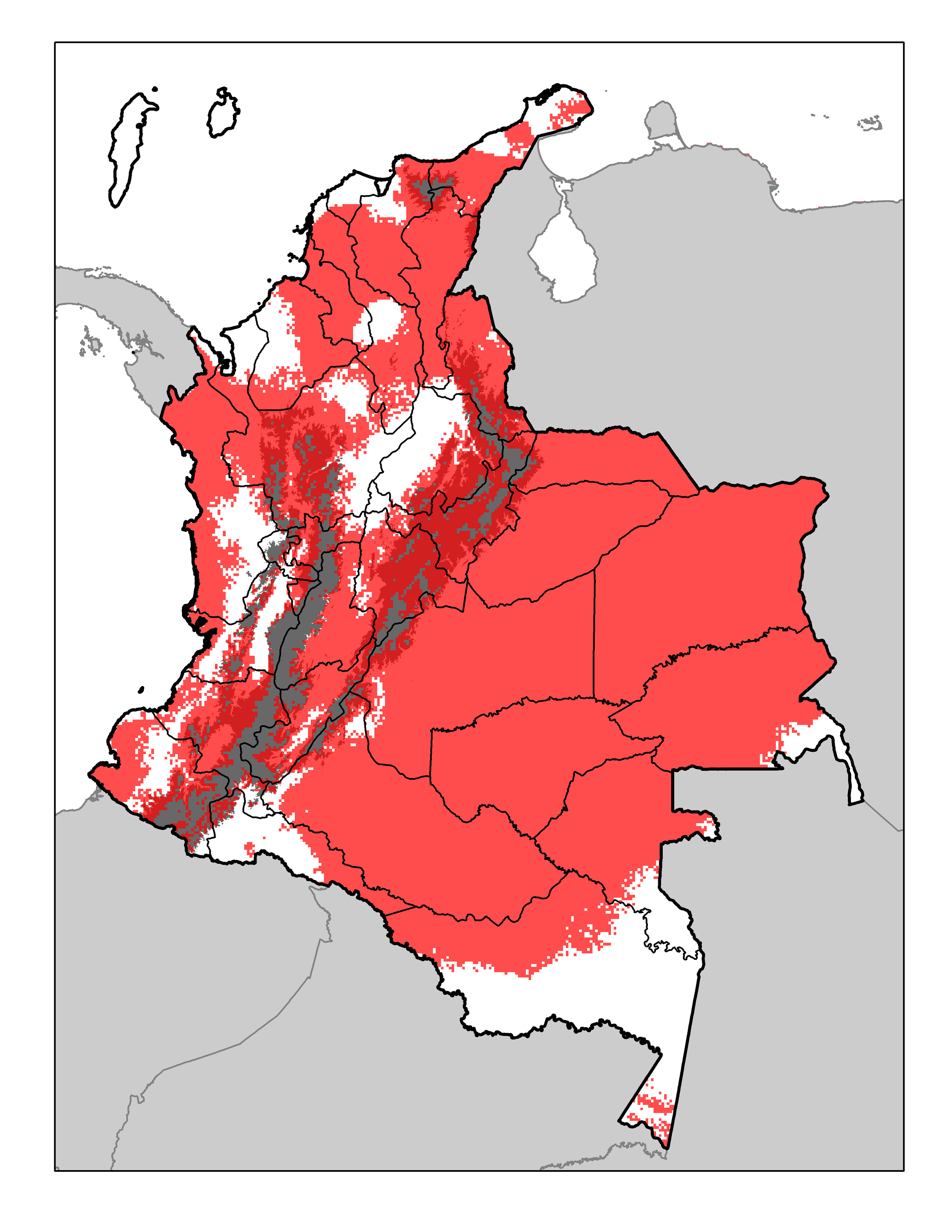

Supplement: S3 File — A. current conditions; B. RCP 2.6 scenario by 2050; C. RCP 8.5 scenario by 2050; D. RCP 2.6 scenario by 2070; E. RCP 8.5 scenario by 2070. The maps were built using the free and open source QGIS software version 3.10.11 (https://www.qgis.org/en/site/about/index.html) and shapefiles were obtained from the free and open source DIVA-GIS site (https://www.diva-gis.org/gdata). (ZIP) [file pntd.0008212.s003.zip › S3_Fig_B.tif]

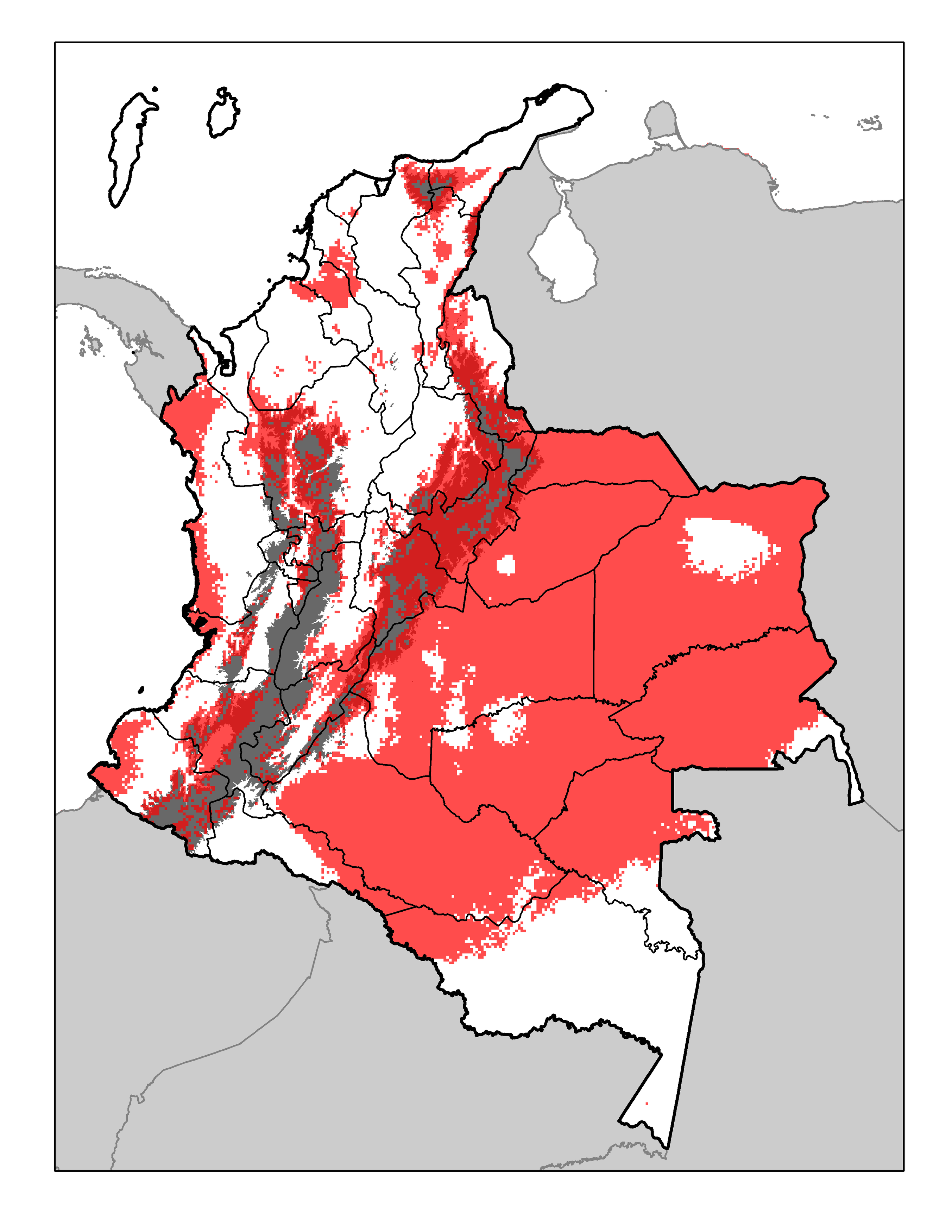

Supplement: S3 File — A. current conditions; B. RCP 2.6 scenario by 2050; C. RCP 8.5 scenario by 2050; D. RCP 2.6 scenario by 2070; E. RCP 8.5 scenario by 2070. The maps were built using the free and open source QGIS software version 3.10.11 (https://www.qgis.org/en/site/about/index.html) and shapefiles were obtained from the free and open source DIVA-GIS site (https://www.diva-gis.org/gdata). (ZIP) [file pntd.0008212.s003.zip › S3_Fig_C.tif]

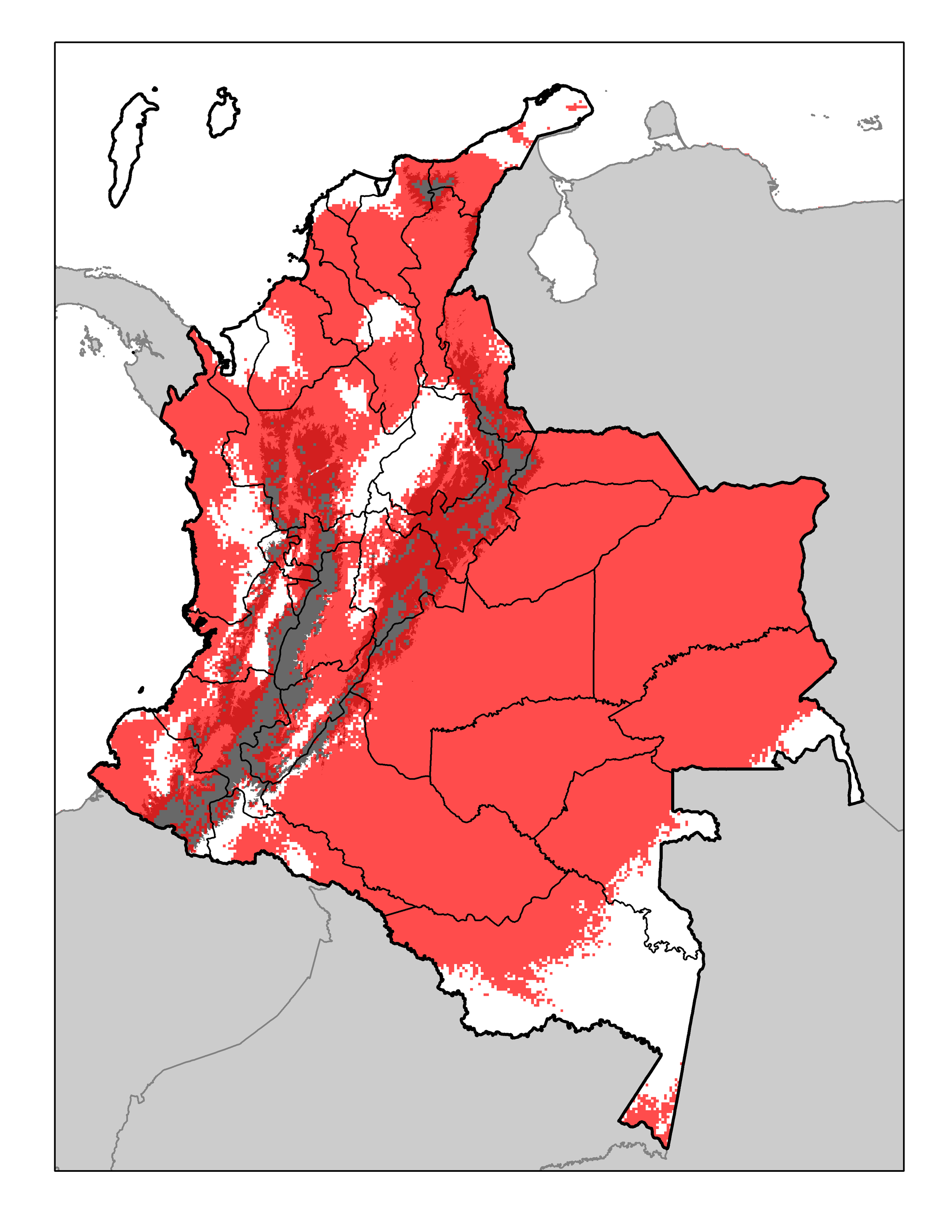

Supplement: S3 File — A. current conditions; B. RCP 2.6 scenario by 2050; C. RCP 8.5 scenario by 2050; D. RCP 2.6 scenario by 2070; E. RCP 8.5 scenario by 2070. The maps were built using the free and open source QGIS software version 3.10.11 (https://www.qgis.org/en/site/about/index.html) and shapefiles were obtained from the free and open source DIVA-GIS site (https://www.diva-gis.org/gdata). (ZIP) [file pntd.0008212.s003.zip › S3_Fig_D.tif]

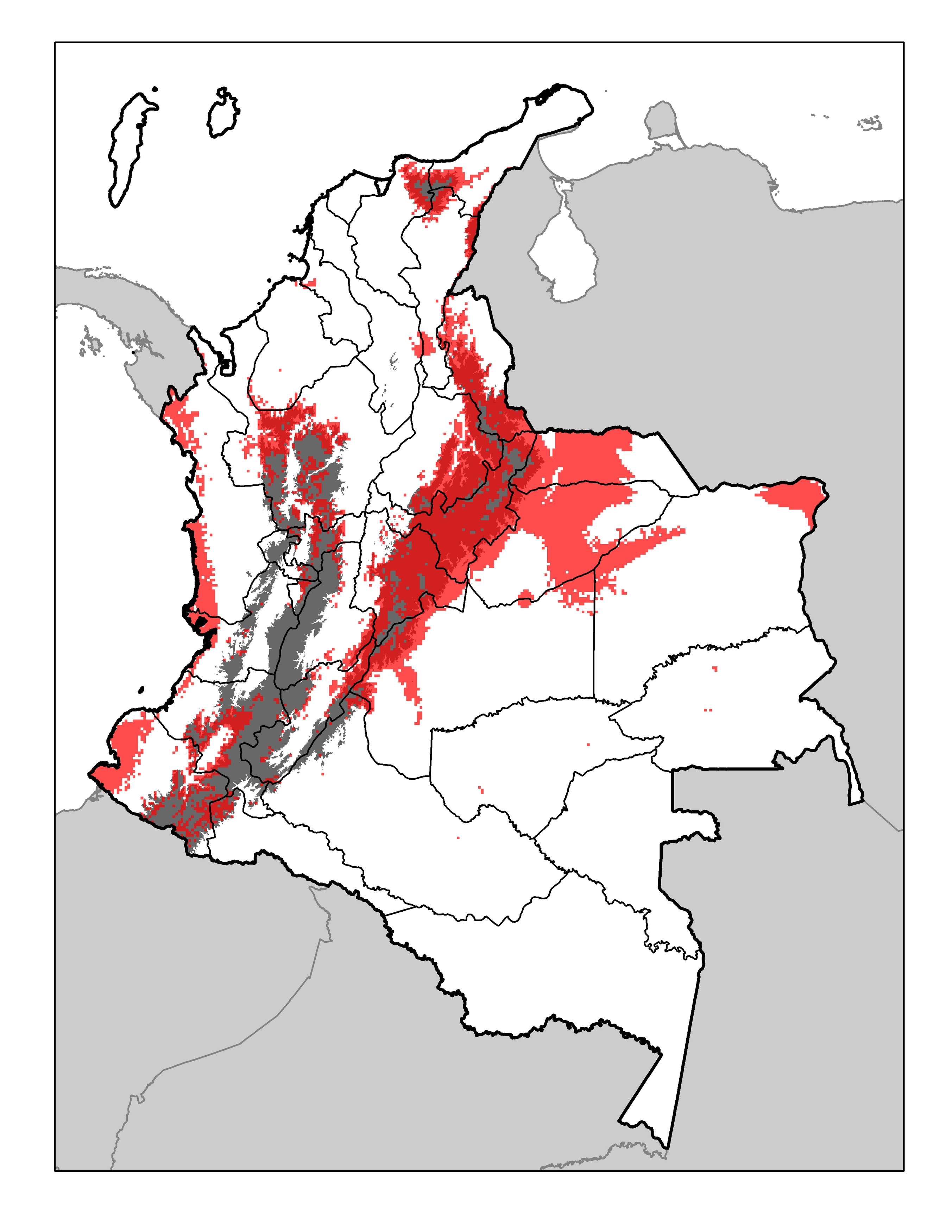

Supplement: S3 File — A. current conditions; B. RCP 2.6 scenario by 2050; C. RCP 8.5 scenario by 2050; D. RCP 2.6 scenario by 2070; E. RCP 8.5 scenario by 2070. The maps were built using the free and open source QGIS software version 3.10.11 (https://www.qgis.org/en/site/about/index.html) and shapefiles were obtained from the free and open source DIVA-GIS site (https://www.diva-gis.org/gdata). (ZIP) [file pntd.0008212.s003.zip › S3_Fig_E.tif]

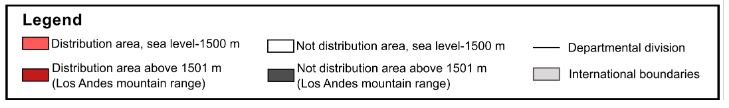

Supplement: S3 File — A. current conditions; B. RCP 2.6 scenario by 2050; C. RCP 8.5 scenario by 2050; D. RCP 2.6 scenario by 2070; E. RCP 8.5 scenario by 2070. The maps were built using the free and open source QGIS software version 3.10.11 (https://www.qgis.org/en/site/about/index.html) and shapefiles were obtained from the free and open source DIVA-GIS site (https://www.diva-gis.org/gdata). (ZIP) [file pntd.0008212.s003.zip › S3_Legend.JPG]
